# Trajectories of Pain Intensity Over 1 Year in Adults With Disabling Subacute or Chronic Neck Pain

**DOI:** 10.1097/AJP.0000000000000727

**Published:** 2019-06-04

**Authors:** Oscar J. Pico-Espinosa, Pierre Côté, Sheilah Hogg-Johnson, Irene Jensen, Iben Axén, Lena W. Holm, Eva Skillgate

**Affiliations:** * Musculoskeletal and Sports Injury Epidemiology Center; ‡ Unit of Intervention and Implementation Research for Worker Health, Institute for Environmental Medicine, Karolinska Institute; § Naprapathögskolan—Scandinavian College of Naprapathic Manual Medicine, Stockholm, Sweden; † Faculty of Health Sciences and UOIT-CMCC Centre for the Study of Disability Prevention and Rehabilitation, University of Ontario Institute of Technology, Toronto, ON, Canada

**Keywords:** neck pain, trajectories, latent class mixed model, course, follow-up, chronic pain, persistent pain, pain progression, clusters

## Abstract

**Objectives:**

The objectives of this study were to describe the 1-year trajectories of disabling subacute or persistent neck pain and to investigate whether baseline age, sex, pain characteristics, and depressive symptoms are associated with such trajectories.

**Study Design and Setting:**

Participants (n=617) included in a randomized controlled trial provided weekly pain intensity ratings by responding to text messages over 1 year. We used latent class mixed model analyses to identify clusters of individual trajectories. Thereafter, we used logistic regression to determine the association between baseline age, sex, pain characteristics, depressive symptoms and treatment, and trajectories of neck pain.

**Results:**

Six different clusters of trajectories were identified. Most participants (73%) followed a trajectory of decreasing pain throughout follow-up. The remaining experienced unfavorable trajectories: persistent pain of high intensity (22%) and slightly (3%) or highly (2%) fluctuating levels of pain reaching high levels of pain intensity. Pain intensity at baseline: odds ratio (OR): 3.76 (95% confidence interval [CI]: 2.49-5.68), depressive symptoms: OR: 3.46 (95% CI: 2.01-5.95), younger age: OR: 2.29 (95% CI: 1.48-3.54), female sex: OR: 1.51 (95% CI: 1.01-2.26), and sudden onset of pain: OR: 1.74 (95% CI: 1.13-2.69) were associated with unfavorable trajectories.

**Conclusions:**

Most individuals with disabling subacute or chronic neck pain show improvement in pain intensity over a year. However, a quarter present unfavorable trajectories. High pain intensity at baseline, depressive symptoms, younger age, female sex, and sudden onset of pain are factors associated with unfavorable trajectories.

## Abstract 2

Supplemental Digital Content is available in the text.

Neck pain has been one of the leading causes of disability worldwide in the past decades.^1^,^2^ The 1 month prevalence of neck pain among adults has been estimated to be between 15% and 45%^3^ and the annual prevalence between 12% and 72%.^3^,^4^ Current evidence suggests that around three quarters of individuals with neck pain will experience another episode between 1 and 5 years later.^5^ Factors associated with poorer prognosis are female sex, older age, concomitant psychosocial pathology, and radicular symptoms.^4^

Courses of neck pain have been described among individuals with prevalent pain at baseline in terms of their pre-post status: resolution, improvement, aggravation, and persistence according to the staging system of the chronic pain questionnaire, showing that 10% become worse, a third improve, 37% persist in the same stage, and a similar percentage reaches resolution.^6^ Such comparisons of proportions over time have also been used to study the course of low back pain.^7^ Data from the HUNT cohort showed a natural decrease in average pain intensity during the first 3 months of follow-up among those who reported some degree of neck/shoulder or back pain at baseline.^8^

There is growing interest on the topic of neck pain course,^9^ specifically on the identification of clusters, accompanied by their comparison in terms of clinically relevant characteristics. The underlying assumption is that nonspecific neck pain is a heterogeneous condition in which various factors interplay. Such factors span from the cultural context and psychosocial characteristics to clinical characteristics such as variability in pain, qualities of pain, and presence of associated conditions such as sleep and fatigue, as stated in the biopsychosocial model of health.^10^,^11^ Thus, such heterogeneity might be reflected in differences in patients’ pain courses^6^ and even in their response to treatment.

Former research studies have used different techniques such as k-means,^12^,^13^ latent class analysis,^14^ or latent class growth analysis,^15^ which are based on the shape of trajectories of pain. Such approaches allow the identification of clinically relevant subgroups and a further exploration of baseline predictive factors characterizing each of them. Latent class mixed modelling (LCMM) is a method that can be used to identify clusters of trajectories. It is based on a Structural Equation Modelling framework and assumes that variability between participants within a cluster exists, allowing for heterogeneity of courses within each generated class.^16^ The output from this type of analysis is a classification of individuals into different groups/clusters on the basis of observed data, representing different trajectories over time.

The main aim of this study was to determine the 1-year pain trajectories of individuals with disabling subacute or persistent neck pain enrolled in a clinical trial. A second aim was to describe the association between age, sex, duration of neck pain, type of onset of neck pain, neck pain intensity, depressive symptoms and treatment arm, and the observed 1-year trajectory patterns. We hypothesized that there would be clusters of favorable (reductions in pain over time) and unfavorable (stable or worsening pain over time) trajectories. We also hypothesized that women, older age, longer duration of pain, insidious onset, higher pain intensity at baseline, and the presence of depressive symptoms would be associated with unfavorable trajectories.

## METHODS

The Stockholm Neck Trial (STONE, Trial registration number: ISRCTN01453590. Registered July 3, 2014) recruited 621 participants from the general population with nonspecific disabling neck pain at enrollment (at least 30 days of duration) and regardless of previous episodes of pain.^17^ The study was advertised in local newspapers in large public companies in Stockholm. Interested individuals contacted a study coordinator who screened for eligibility and referred participants to a study therapist for a clinical assessment. Participants were included if they rated their pain intensity ≥2/10 and disability due to pain ≥1/10 on Numerical Rating Scales (NRS) based on questions adapted from the Chronic Pain Questionnaire, which measured current pain intensity, average pain intensity in the past month, and worst pain intensity in the past month, and pain-related disability related to general daily activities, pain-related disability related to work, and pain-related disability related to social activities.^18^ Participants were randomly assigned to 1 of 4 different groups: (1) Swedish massage, (2) strengthening and stretching exercises, (3) a combination of (1) and (2), and (4) advice to stay active.^17^ Blinding was not possible due to the nature of the interventions. Up to 6 sessions of therapy were delivered over 6 weeks (up to 3 for advice to stay active), and participants completed questionnaires at baseline, 7 weeks, 3 months, 6 months, and 12 months. A thorough description of the procedures of the trial is available in the study protocol.^19^

Informed consent was obtained from all individual participants included in the study, and all procedures performed were in accordance with the ethical standards of the Regional Ethic Committee in Stockholm (Dnr: 2014/755-31/3) and with the 1964 Helsinki declaration and its later amendments or comparable ethical standards.

The mean age of the study population was 46 years, 69% were women, 58% had had neck pain for at least 1 year, 67% reported a slow onset of pain, and 77% took at least 1 form of pain management medication.

### Follow-Up

Besides answering questionnaires, information about average neck pain intensity was collected via text message (SMS) every week over 1 year, yielding 52 repeated measurements (53 for some individuals, as 2016 was a leap year). The question was: *How intense has your neck pain been on average in the past week? Enter a number between 0 (no pain) to 10 (worst pain imaginable)*, and the answers were automatically stored in an electronic database. This methodology has been used by other authors, showing high feasibility and excellent compliance.^20^ Furthermore, a study coordinator monitored the data collection every week and sent reminders in case of missing responses, first with SMS and then by a telephone call if needed. The response rate was 92%, calculated as the total number of pain reports via SMS (29,934) over the total requests sent (32,654). We did not collect information on reasons for dropping out of the study.

### Statistical Analysis

The resulting data set was extracted from the electronic platform and prepared for analyses. Spaghetti plots of pain over time were generated to visually inspect the data. LCMM^21^ was applied considering linear, quadratic, and cubic models. There were issues with model convergence when cubic parameters were included, and therefore only linear and quadratic models were ultimately fitted and compared. A stepwise procedure was followed, in which a model with only 1 class was performed first, and then parameter estimates from that model were used as initial values in subsequent models with increasing numbers of clusters, and the BIC (Bayesian Information Criterion) served as an indicator of goodness of fit, with lower values indicating better fit.^22^ After choosing the best fitting model, probabilities of class membership were obtained for each individual, and individuals were assigned to the class of highest probability. The selected model included a dichotomized variable on depressive symptoms, from the Hospital Anxiety and Depression Scale,^23^ as membership predictor. This variable was chosen after visual inspection of the stratified average pain, wherein an important difference was judged to be present; additionally, goodness of fit of the model improved when this variable was added. Analyses were performed with the hlme—function of the R-lcmm package.^21^ To facilitate interpretation of the trajectory clusters, the fitted curves of the trajectories from the final model and the mean pain intensity over time were plotted.

Subsequently, we obtained descriptive statistics of the characteristics of participants in each cluster of trajectories. Such clusters were further classified into 2 main classes based on visual inspection: “favorable” and “unfavorable.” The favorable class was defined as mean pain intensity following a constant decrease over the follow-up period or a decrease in mean pain intensity preceded or followed by stable values. In contrast, the unfavorable class was defined as increases in pain intensity, constant higher levels of pain intensity, or fluctuating courses reaching the upper half of the pain intensity scale at the follow-up. Univariable logistic regression analyses were run to assess associations between baseline factors specified a priori: age at baseline; sex; duration of neck pain categorized as 1 to 3, 3 to 6, 6 to 12, or >12 months; type of onset of neck pain categorized as insidious, sudden, or unsure; neck pain intensity dichotomized as 0 to 5/10 and 6 to 10/10; depressive symptoms measured with the hospital and anxiety scale and dichotomized using 8/9 as cutoff point;^23^ treatment arm; and trajectory class. These baseline factors were chosen, as they are usually considered in encounters with patients with subacute and chronic neck pain. Subsequently, multivariable logistic regression models for each of these factors were performed for 1 factor at a time and adjusting for other variables from the list if considered appropriate on the basis of previous knowledge. The treatment arm was thought of as an effect modifier, and therefore it was not included in any of the other multivariable models. Stata 14.0 was used for that purpose.^24^

Moreover, after observing the magnitude of the odds ratio (OR) for the baseline variables showing the strongest associations, the interaction was examined in the additive scale between baseline pain intensity and depressive symptoms with regard to the risk of showing an unfavorable cluster trajectory. We calculated the relative excess risk due to interaction of trajectory cluster membership,^25^ wherein 0 indicates no interaction, >0 indicates positive interaction (the effect of both exposures combined is larger than the sum of them), and <0 indicates negative interaction.^26^

As a sensitivity analysis to test whether different clusters would be observed depending on levels of neck pain intensity and neck pain duration at baseline, we re-ran the LCMM analysis for each of the following strata of study participants: low intensity at baseline (NRS ≤3), middle intensity at baseline (NRS 4 to 6), and high intensity at baseline (NRS ≥7), and in the strata of pain duration (less than and at least 1 year).

## RESULTS

We used data from 617 participants after excluding 2 who did not reply to any SMS and another 2 who requested their information to be removed from the study. Participants contributed with a median of 53 weeks (interquartile range: 52 to 53 wk) and a mean of 48.4 (SD 12.4) weeks.

**FIGURE 1:**
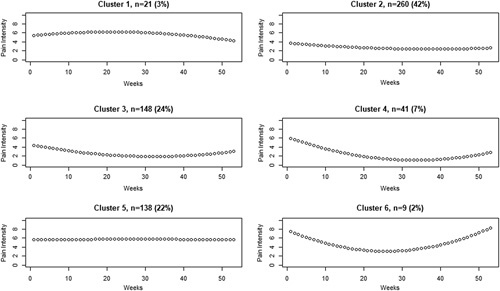
Latent classes of neck pain courses over 1 year retrieved from the latent class mixed modeling analysis and the number of individuals in each class.

**FIGURE 2:**
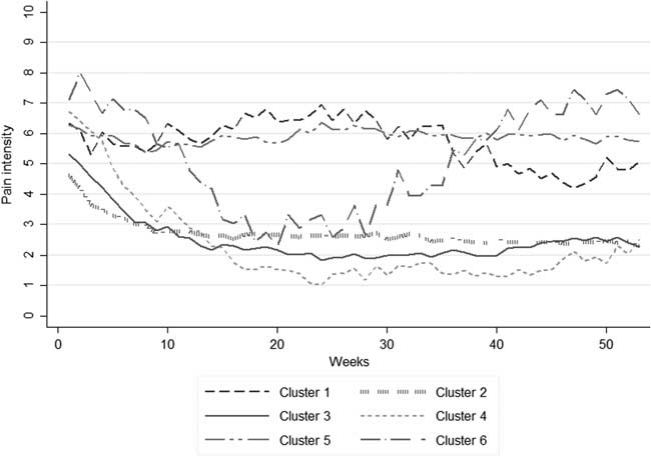
Mean pain intensity over time by clusters generated with the latent class mixed modeling analysis.

Table 1 shows the BIC, posterior probabilities, and number of participants assigned to each tested class for models including from 1 to 7 clusters. The model with 6 different clusters of trajectories had the lowest BIC, indicating best goodness of fit and was therefore chosen for further analyses. In addition, high posterior probabilities indicated good differentiation between clusters. Figure 1 shows the fitted clusters retrieved from the LCMM analysis (individual trajectories within each cluster are presented in spaghetti plots in Annex A, Supplemental Digital Content 1, http://links.lww.com/CJP/A578), and Figure 2 presents the mean pain intensity over time for the 6 clusters in 1 single graph.

**TABLE 1:**
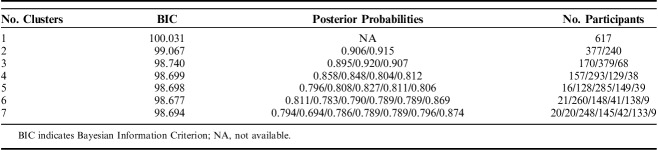
BIC, Posterior Probabilities, and Number of Participants For Each Tested Model

Cluster 1: “slightly fluctuating” (3%, n=21) was characterized by a slight increase during the first half of the follow-up followed by a decrease and a further slight increase at the end. Cluster 2: “small improvement” was the most common one (42%, n=260) and consisted of individuals with moderate pain intensity at baseline showing improvement during the first part of the follow-up and subsequent constant low pain intensity. Cluster 3: “moderate improvement” was the second most common (24%, n=148) following a similar pattern as cluster 2, but with a larger decrease in pain. Cluster 4: “large improvement” (7%, n=41) showed a constant large decrease in pain during the first 6 months followed by constant low levels of pain. Cluster 5: “persistent” (22%, n=138) showed constantly high levels of pain intensity with minimal variations. Cluster 6: “largely fluctuating” (2%, n=9) was the trajectory with the smallest number of individuals and showed the highest baseline pain intensity, a large decrease of pain in the first part of the follow-up, followed by an equally large and steady increase of pain intensity.

Table 2 shows participants’ characteristics at baseline for each cluster. Depressive symptoms were more common among those in clusters 5 and 6, and cluster 5: “persistent pain” had a higher proportion of women than the others.

**TABLE 2:**
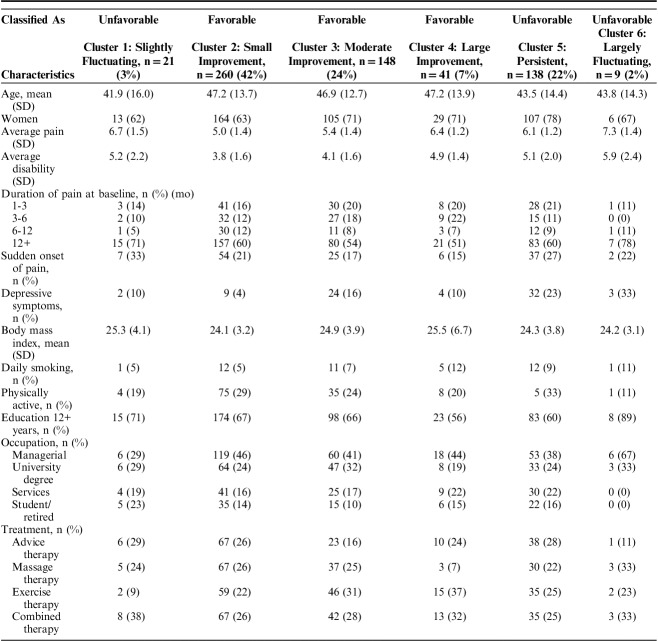
Patient Characteristics at Baseline by Cluster

Clusters 2, 3, and 4 were labeled as “favorable,” whereas clusters 1, 5, and 6 were labeled as “unfavorable.” Table 3 shows the ORs from logistic regression of baseline variables associated with favorable versus unfavorable courses. Female sex: OR: 1.52 (95% confidence interval [CI]: 1.02-2.27), young age: OR: 2.30 (95% CI: 1.49-3.55), sudden onset of pain OR: 1.74 (95% CI: 1.13-2.69), depressive symptoms OR: 3.46 (95% CI: 2.01-5.95), and higher pain intensity: OR: 3.76 (95% CI: 2.49-5.68) were associated with worse trajectories.

**TABLE 3:**
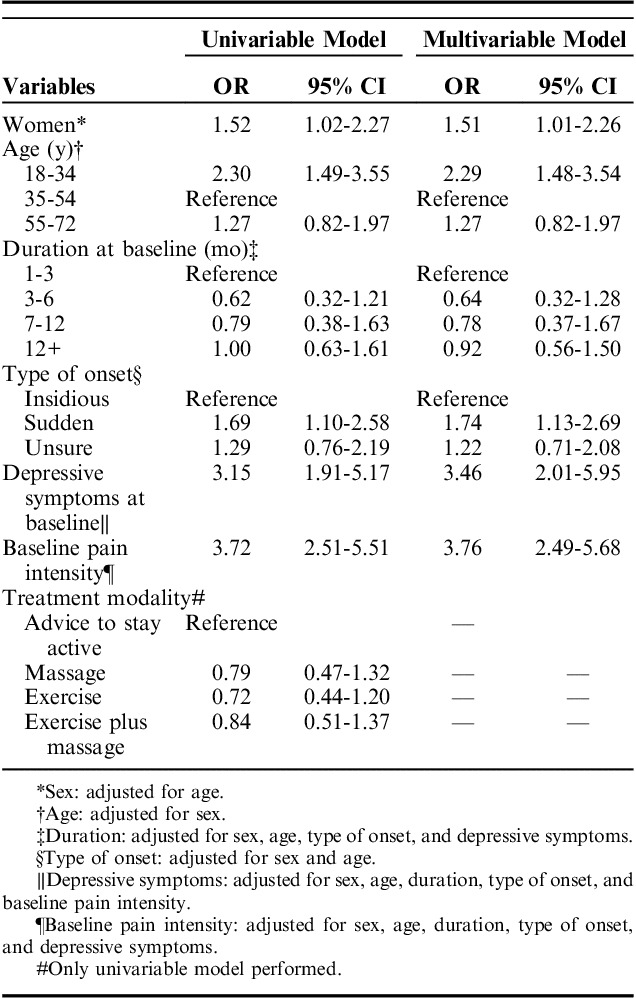
Odds Ratio (OR) and 95% Confidence Intervals (95% CIs) of Selected Baseline Variables For Belonging to Unfavorable Versus Favorable Trajectories (Reference)

**FIGURE 3:**
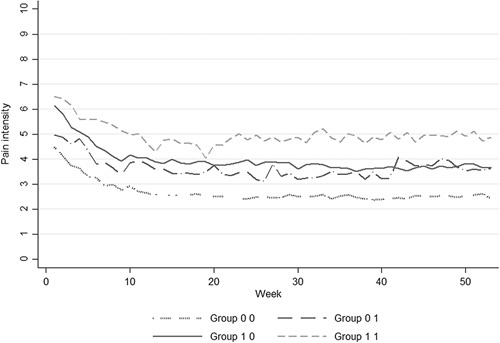
Course of pain intensity over 1 year by depressive symptoms and pain intensity at baseline. Group 0 0: baseline pain ≤5/10 and no depressive symptoms (n=262, 42%), group 0 1: baseline pain ≤5/10 with depressive symptoms (n=33, 5%), group 1 0: baseline pain ≥6/10 and no depressive symptoms (n=281, 46%), and group 1 1: baseline pain ≥6/10 with depressive symptoms (n=41, 7%).

Participants with both high pain intensity and depressive symptoms at baseline had higher levels of pain intensity over the follow-up compared with those with only 1 or none of these conditions (Fig. 3). However, in the analysis of interaction, the relative excess risk due to interaction was −0.47 (95% CI: −2.55 to 1.62), indicating a nonsignificant negative interaction.

In the sensitivity analysis, less number of clusters were generated in the subsets of the population given a smaller sample size (data not shown). However, we considered that, overall, the 6 clusters presented here represent the ones generated in each subset of the data.

## DISCUSSION

We chose a model containing 6 different well-differentiated clusters of trajectories. The majority of individuals (73%) were classified in one of the favorable trajectories, showing a decrease of pain intensity of between 2 and 5 U in the NRS during the first part of the follow-up. A similar proportion of improvement has been reported previously by others.^5^ The rest of the participants formed what we called unfavorable clusters, which we judged as clinically challenging and worthy of further study as a class compared with the other clusters with a favorable course of neck pain. Higher pain intensity at baseline and having depressive symptoms were the factors showing the strongest association with having unfavorable trajectories of neck pain. Besides, female sex, sudden pain onset, and younger age were associated with higher odds of reporting unfavorable trajectories. Most of these factors have been found to be of importance in the prognosis of neck pain.

Nevertheless, younger age has been associated with better outcomes, which is discordant with our findings.^5^ We believe that younger age might rather be a proxy for personal and environmental factors (such as living conditions or work characteristics), which may contribute to worse outcomes. Hypotheses about the impact of age and other sociodemographic and/or psychosocial factors on trajectories need to be studied in the future.

Three previously published studies have used clustering techniques for the study of the course of neck pain. Walton et al^27^ reported 2 different clusters, 1 showing a slight increase and the other showing an important decrease. However, the short follow-up (4 weeks) and the small sample size (n=50) limited the identification of different clusters. Ailliet et al^15^ collected a larger number of individuals with neck pain and identified 4 different classes. Similar to our findings, the majority of their participants were classified in courses characterized by a recovery from a mild baseline pain with a small proportion showing larger decreases of pain. In addition, some individuals showed an increase in pain, which corresponds with the increase we observed in “cluster 6: largely fluctuating” during the first 6 months of our follow-up. Likewise, Hallman et al^28^ collected monthly measures of pain on workers—with and without pain—and identified 6 different clusters, showing patterns comparable to ours, such as high intensity, persistent, and others with different degrees of decrease over time depending on baseline level.

Studies of the course of low back pain, a condition very often coexisting with and related to neck pain, have revealed similar patterns to those identified here. Downie and colleagues studied the course of acute low back pain, showing that, after 12 weeks, 7 of 10 participants had achieved complete recovery, around 5% reported constantly high pain intensity, and the rest showed either a fluctuating pain intensity or incomplete recovery. Those with persistent pain tended to be on sick leave and to report higher pain intensity at baseline, greater pain duration, and worse quality of life.^29^ Kongsted et al^9^ also examined thoroughly various modeling strategies to identify courses of chronic low back pain, showing that between 4% and 12% corresponded to a “severe ongoing” course of pain, with an average pain intensity of 8.1/10. They also identified a relapse cluster, which would correspond to our cluster 6: “largely fluctuating,” and the rest corresponding to different degrees of recovery generally occurring at the beginning of the follow-up period, as seen in our results. In addition, they identified depression and longer duration as predicting factors of the classes.^9^

We did not see a clear distinction between the fast and slow improvers, as previously described by other authors.^12^ On average, the improvement in our sample occurred between the 16th and 20th week in all classes.

### Strengths and Limitations

This study has strengths worth highlighting. First, this is among the largest studies aiming to describe the course of subacute and chronic neck pain. Second, the response rate was very high, and, together with the choice of LCMM as the statistical method, it was possible to use all the information available. Third, the follow-up was long enough to see important shifts in the trajectories: for instance, creating clusters based on the first 6 months only, would have led to an incomplete picture, as, after that time, individuals either went back to their original higher levels of pain such as cluster 6: “largely fluctuating,” or reached a plateau instead of continuing decreasing pain such as in cluster 4: “large improvement.” Fourth, we included a heterogeneous group of self-selected individuals from the general population, and the clusters identified by us correspond to those reported in previous publications, which indicates that our findings can be generalized to other populations. Last, as indicated, the clusters generated in the sensitivity analyses are well represented in the ones observed in the main analysis.

In contrast, 1 of the weaknesses we observed is that some of the trajectories had very few individuals (cluster 6: n=9; cluster 1: n=21), making cluster-specific regression analyses inappropriate due to lack of power. Instead, we opted for grouping clusters in favorable and unfavorable classes to identify associations with relevant baseline characteristics. In addition, although we performed analyses in multivariable models for the associations between baseline characteristics and an unfavorable trajectory, we might have a problem with residual and unmeasured confounding in these results. Last, it is likely that a different exposure categorization had led to slightly different results: for instance, by using either a continuous variable for duration of pain or more detailed categories.

### Implications

The current findings indicate that persistent nonspecific neck pain might be a heterogeneous condition in terms of its course. Moreover, certain personal baseline characteristics (pain intensity, sex, age, psychological distress, and type of onset) seem to influence this course. The participants included in the study underwent a cycle of therapies at the beginning of the randomized controlled trial. Despite this, more than a fifth of them showed unfavorable patterns characterized by no or minimal improvement in pain intensity. It is possible that some personal baseline characteristics have a stronger influence on the course of the condition than the treatment provided, whereas participants with favorable patterns improved regardless of which therapy they received. It is also possible that individuals with neck pain, in particular, those with a worse course of the disease, may require therapeutic approaches that target >1 dimension of health, as the biopsychosocial model proposes, or that these patients are unresponsive to all treatments.^11^ Clinicians should be aware of this and communicate to their patients that no improvement in pain intensity or very minimal changes over 1 year may be a probable outcome.

## CONCLUSIONS

Most individuals with disabling subacute or chronic neck pain show improvement in pain intensity over 1 year. However, a quarter present unfavorable trajectories, following either a fluctuating or a persistent pattern of pain over time despite undergoing a cycle of therapies for pain control. High pain intensity at baseline, depressive symptoms, younger age, female sex, and sudden onset of pain are factors associated with unfavorable trajectories of neck pain in this study.

## Supplementary Material

SUPPLEMENTARY MATERIAL

Supplemental Digital Content is available for this article. Direct URL citations appear in the printed text and are provided in the HTML and PDF versions of this article on the journal's website, www.clinicalpain.com.
