# Supplementary figures and images for: Trajectories of Pain Intensity Over 1 Year in Adults With Disabling Subacute or Chronic Neck Pain
Source: Clin J Pain. 2019 Jun 4;35(8):678–85. doi: 10.1097/AJP.0000000000000727 (PMC6615962; doi:10.1097/AJP.0000000000000727)

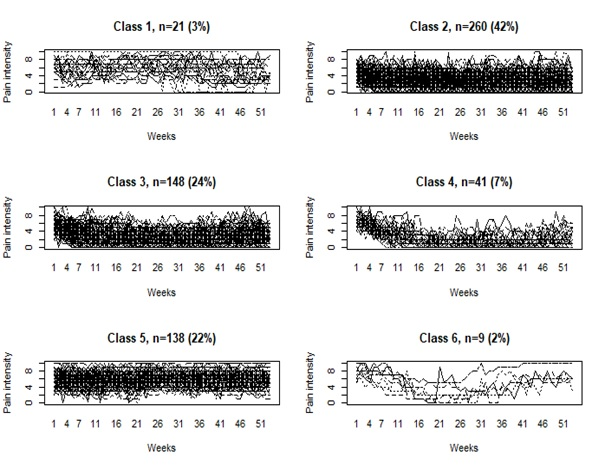

Supplement: SUPPLEMENTARY MATERIAL [file ajp-35-678-s001.tif]
